# Duration and Continuity of Medicaid Enrollment Before the COVID-19 Pandemic

**DOI:** 10.1001/jamahealthforum.2022.4732

**Published:** 2022-12-16

**Authors:** Leighton Ku, Isabel Platt

**Affiliations:** 1 Center for Health Policy Research, Milken Institute School of Public Heath, George Washington University, Washington, DC

## Abstract

**Importance:**

COVID-19 relief legislation created a temporary moratorium on Medicaid disenrollment, but when the public health emergency ends, states will begin to “unwind” Medicaid enrollment. Prepandemic data shed light on factors that can affect Medicaid coverage stability.

**Objective:**

To assess factors associated with the duration and continuity of Medicaid enrollment.

**Design, Setting, and Participants:**

In this cross-sectional analyses of a Medicaid data set for 2016 that was released by the Agency for Healthcare Research and Quality in June of 2022, we analyze a nationally representative data set of 5.7 million persons, weighted to represent 70 million Medicaid beneficiaries in 2016. We focus on 22 million nondisabled, nonelderly adults for this analysis. The data were analyzed between July and September of 2022.

**Main Outcomes and Measures:**

The main outcomes were the average months of Medicaid enrollment in 2016 and the probability of *churning*, defined as a break in coverage between 2 periods of enrollment during the calendar year. We compared these outcomes by eligibility category, state, demographic characteristics, and key Medicaid policies, including whether the state expanded Medicaid and whether it used ex parte reviews (automated reviews of other administrative data to reduce renewal paperwork burdens).

**Results:**

In this cross-sectional analysis, we analyze a nationally representative Medicaid data set of 5.7 million persons, weighted to represent 70 million Medicaid beneficiaries in 2016, released by the Agency for Healthcare Research and Quality in June of 2022. The analysis focused on nonelderly, nondisabled adults (aged 18-64 years) with a weighted population size of 22.7 million, of which 18.4% were Black, 19.2% were Latino, 39.5% were White, 7.3% were other/Asian/Native American, and 15.5% had unknown race. Multivariable regression analysis indicated that those living in states that expanded Medicaid but did not use ex parte reviews had longer average duration (0.31 months longer; 95% CI, 0.03-0.59) and lower risk of churning(odds ratio [OR], .40; 95% CI, 0.39-0.40), whereas those living in nonexpansion states that used ex parte reviews had lower odds of churning (OR, .68; 95% CI, 0.66-0.70) but also had shorter average duration (3.1 months shorter; 95% CI, −3.4 to −2.8). Those living in expansion states that used ex parte reviews also had reduced churning (OR, .83; 95% CI, 0.82-0.85). The average duration varied widely by state, even after adjustments for demographic and state policy factors.

**Conclusions and Relevance:**

If state Medicaid programs revert to prepandemic policies after the temporary moratorium ends, Medicaid coverage, particularly for nondisabled, nonelderly adults, is likely to become less stable again. Medicaid expansions are associated with improved continuity, but ex parte review may have a more complex role.

## Key Points

**Question:**

How stable was the continuity of Medicaid enrollment from 2016 to before the COVID-19-related moratorium on disenrollment, and what factors contributed to stability?

**Findings:**

This cross-sectional analysis of nationally representative Medicaid data on 5.7 million persons show that Medicaid expansion was associated with less *churning* (defined as one or more breaks in coverage) and longer duration of coverage, whereas the use of ex parte reviews was associated with less churning but shorter duration of coverage. Even after controlling for demographic and state policy factors, there were very large interstate differences in Medicaid continuity.

**Meaning:**

After the federal COVID-19 public health emergency declaration expires and the disenrollment moratorium ends, states should consider adopting Medicaid policies that stabilize the continuity of coverage.

## Teaser

This cross-sectional study uses Medicaid data to assess factors associated with the duration and continuity of Medicaid enrollment.

## Introduction

Until 2020, enrollment in Medicaid had to be renewed at least every 12 months and was often subject to periodic checks within a certification period. Changes in income or eligibility category, such as age or pregnancy status, or paperwork barriers in the renewal process often led to the loss of Medicaid benefits and *churning* (defined as a break in coverage between 2 periods of enrollment during the calendar year) as people exited then reentered the program.^1^ The primary forms of health insurance coverage in the US are more stable: Medicare enrollment is essentially continuous after people become senior citizens and employer-sponsored coverage usually lasts as long as a person is enrolled in the same job. Historically, Medicaid coverage has been more volatile.

Disruptions to Medicaid coverage can have adverse effects, including interruptions in access to regular care and medications and increases in preventable hospital admissions.^1,2,3,4,5,6,7^ Disruptions also increase average monthly Medicaid costs per enrollee, in part because the loss of primary care or medications during the gap could lead to higher emergency or inpatient hospital use.^8,9^ Churning can also increase Medicaid administrative costs, both for states as well as for managed care plans.^1^

In February of 2020, a COVID-19 relief bill, the Families First Coronavirus Response Act, increased the federal Medicaid matching rate to provide fiscal relief to states during the pandemic and required states to maintain Medicaid enrollment during the federal COVID-19 public health emergency declaration to help maintain insurance coverage and prevent economic hardship during the pandemic.^10^ New applicants could join Medicaid but enrollees are not terminated unless they move across state lines or voluntarily drop coverage. After the public health emergency ends, states will have about 12 months to redetermine eligibility for all their Medicaid enrollees and terminate coverage for those no longer eligible. As of November 2022, the public health emergency has been extended through January 2023.^11^

Since the moratorium on disenrollments began in February 2020, Medicaid caseloads grew by over 15 million people. The “unwinding” may trigger large reductions in the year after the public health emergency ends.^12,13^ The Centers for Medicare & Medicaid Services (CMS) are urging states to take steps to carefully redetermine Medicaid eligibility and to help those losing Medicaid transition to other forms of insurance.^10,14,15^

This study analyzed the continuity of Medicaid enrollment in 2016, before the moratorium began, to examine factors affecting continuity in the absence of the moratorium. We describe the variation in continuity and factors associated with enrollment duration and churning. This study focuses on nondisabled, nonelderly adult enrollees, the group with the least stable enrollment.^8^

## Methods

### Data

This study was primarily based on analyses of data from a novel, nationally representative database, released by the Agency for Healthcare Research and Quality (AHRQ) in June of 2022: the Synthetic Healthcare Database for Research (SyH-DR).^16^ The SyH-DR compiles claims data for Medicaid, Medicare, and commercial insurance, weighted to be nationally representative of health care use in the US. It represents the agency’s initial effort to create an all-payer database. This study was determined exempt by the George Washington University institutional review board because all SyH-DR data are deidentified and the system uses aggregation and “synthetization” (eg, selective scrambling) of certain data elements to protect the privacy of health data.^17^ These analyses were conducted between July and September 2022.

This study examined data from the Medicaid person file, one part of SyH-DR. This is a large sample of data from the Transformed Medicaid Statistical Information System (T-MSIS). It includes data for 5.7 million Medicaid enrollees drawn from 49 states (all but Arkansas) and the District of Columbia in calendar year 2016, representing 69.8 million total enrollees nationwide who were enrolled in Medicaid for at least 1 month. Data included sex, race/ethnicity (Black, Latino, White, Other/Asian/Native American, and unknown), age (grouped as 0-5, 6-17, 18-24 years, etc), eligibility category, monthly Medicaid enrollment (January through December 2016), and monthly enrollment in a Medicaid managed care organization (MCO). Medicaid eligibility categories included: Medicaid children; Children’s Health Insurance Program children (CHIP); those with disabilities; aged adults; nonelderly, nondisabled, adults enrolled in traditional categories; and adults covered by Affordable Care Act (ACA) Medicaid expansions. None of the data in this specific analysis were synthesized; they reflect actual data in the state Medicaid files, although some variables have been aggregated or simplified to protect privacy.

We merged the SyH-DR data with state Medicaid policies for 2016 reported by the Kaiser Family Foundation, including whether the state used options under the ACA to expand Medicaid eligibility by January 1, 2016, and whether the state reported the use of ex parte reviews, described below.^18^ The eAppendix in the Supplement provides more information about the methods and analyses.

The primary outcomes for this study were (1) the number of months a person was enrolled in Medicaid in calendar year 2016, the total period with access to Medicaid benefits in 2016, and (2) whether a person experienced churning, defined as a break in coverage between 2 periods of enrollment during the calendar year. Churning often reflects problems with the renewal process.^1,13^ Because these individuals were enrolled in Medicaid before and after the break, many were still eligible but lost enrollment due to paperwork burdens that prevented timely renewal. For example, they might not have received a renewal notice because the state did not have their correct address or had difficulty completing the application.

A Medicaid policy designed to reduce churning and the burdens of renewal paperwork is the use of automated ex parte reviews.^19^ By regulation, before a state sends out a renewal notice for certain Medicaid enrollees, it must first review data sources already available that can be used to renew coverage automatically. For example, if a person’s Medicaid certification period expires in March, but SNAP eligibility was determined in January, the SNAP data can be used to renew Medicaid coverage and there is no need to ask for a separate Medicaid renewal application, which reduces the administrative burden of renewals.

### Statistical Analysis

All the analyses use survey weights developed by AHRQ that represent Medicaid beneficiaries at the state and national levels. We first conducted weighted descriptive analyses, seen in Table 1 and Table 2 (and eTables 1 and 2 in the Supplement). To more closely examine factors that affect continuity of adult coverage, we conducted multivariable analyses using ordinary least squares linear regression and logistic regression models, where the outcomes were (1) the number of months of Medicaid enrollment during the year and (2) the odds of churning, defined as a break in coverage between 2 periods of enrollment in the year. In addition to controlling for demographic characteristics (age, sex, and race/ethnicity), we focused on 2 key state policy factors. We included whether the person lived in a state that expanded Medicaid eligibility, a state that reported using ex parte reviews, and the combination of expansion and ex parte policies. We used state fixed effects to control for unmeasured state characteristics (eg, other policy, political, or socioeconomic factors) that differ across states, allowing our analysis to isolate the effects of the demographic and policy variables on our outcomes. We included standard errors clustered at the state level, because the policies are uniform within each state. As discussed in the eAppendix in the Supplement, we could not estimate coefficients for state fixed effects for all states because of multicollinearity or because some states had no breaks in enrollment.

**Table 1.** Weighted Descriptive Data About Medicaid Continuity in 5 403 960 Enrollees in 2016

Category	Enrollees, No.	Mean (95% CI)	
		Months of Medicaid enrollment	Probability of churning
Medicaid children	26.4 Million	10.54 (10.54-10.55)	3.4 (3.4-3.4)
CHIP children	2.3 Million	10.09 (10.08-10.11)	8.0 (7.9-8.1)
Traditional adults	11.6 Million	9.48 (9.47-9.49)	4.2 (4.1-4.2)
Expansion adults	10.9 Million	9.80 (9.80-9.81)	3.8 (3.7-3.8)
Disabled	7.6 Million	11.15 (11.14-11.15)	1.4 (1.4-1.4)
Aged	6.4 Million	11.19 (11.18-11.20)	0.6 (0.6-0.7)

Abbreviation: CHIP, Children's Health Insurance Program.

**Table 2.** Weighted Annual Enrollment and Average Number of Months Enrolled in Medicaid for Nonelderly, Nondisabled Adults in 2016, by State

Variable	Enrolled, No. (1000s)	Mean months (95% CI)
States that expanded Medicaid by January 2016		
Total, expanded	17 127.9	9.83 (9.82-9.84)
Alaska	48.3	9.54 (9.44-9.63)
Arizona	586.5	10.70 (10.67-10.72)
California	4494.3	9.60 (9.59-9.60)
Colorado	436.5	9.96 (9.93-9.99)
Connecticut	339.0	9.93 (9.89-9.97)
Delaware	37.9	8.14 (8.01-8.27)
District of Columbia	88.2	10.67 (10.61-10.74)
Hawaii	100.1	10.72 (10.67-10.78)
Illinois	1074.5	9.83 (9.81-9.85)
Indiana	426.4	8.46 (8.42-8.51)
Iowa	238.6	9.38 (9.33-9.43)
Kentucky	386.2	10.90 (10.87-10.92)
Maryland	439.6	9.89 (9.85-9.92)
Massachusetts	778.3	9.79 (9.77-9.82)
Michigan	952.8	9.51 (9.49-9.54)
Minnesota	490.5	8.87 (8.83-8.91)
Montana	62.0	8.87 (8.78-8.96)
Nevada	211.4	8.73 (8.68-8.78)
New Hampshire	60.0	8.76 (8.65-8.86)
New Jersey	529.9	9.44 (9.41-9.48)
New Mexico	239.2	10.75 (10.72-10.78)
New York	2150.7	10.58 (10.57-10.59)
North Dakota	26.6	7.77 (7.56-7.99)
Ohio	960.4	10.56 (10.54-10.58)
Oregon	388.3	10.25 (10.22-10.28)
Pennsylvania	904.1	9.10 (9.08-9.13)
Rhode Island	93.1	10.06 (9.99-10.13)
Vermont	38.6	10.34 (10.25-10.44)
Washington	355.8	9.75 (9.71-9.78)
West Virginia	190.1	9.77 (9.72-9.82)
States not expanding Medicaid by January 2016		
Total, not expanded	5166.8	8.96 (8.94-8.97)
Alabama	227.3	8.74 (8.69-8.80)
Florida	960.2	10.07 (10.04-10.09)
Georgia	387.5	7.80 (7.75-7.84)
Idaho	60.6	8.54 (8.41-8.66)
Kansas	88.3	9.36 (9.26-9.46)
Louisiana	656.1	8.95 (8.91-8.99)
Maine	79.8	9.48 (9.38-9.59)
Mississippi	149.3	8.97 (8.90-9.04)
Missouri	32.9	11.21 (11.12-11.30)
Nebraska	56.9	8.19 (8.06-8.32)
North Carolina	426.7	10.23 (10.20-10.26)
Oklahoma	264.6	7.91 (7.85-7.98)
South Carolina	281.5	10.45 (10.41-10.48)
South Dakota	31.0	7.82 (7.64-8.01)
Texas	624.2	7.02 (6.98-7.06)
Utah	114.8	7.61 (7.50-7.71)
Virginia	259.7	9.02 (8.97-9.07)
Wisconsin	451.5	9.03 (8.99-9.07)
Wyoming	13.9	9.43 (9.21-9.65)

This study was deemed exempt from institutional board review by the George Washington University institutional review board because we analyzed deidentified secondary data. It was conducted in accordance with the Strengthening the Reporting of Observational Studies in Epidemiology (STROBE) reporting guideline for cross-sectional studies.^20^

## Results

### Descriptive Data

Table 1 presents weighted descriptive data about the Medicaid enrollees, classified by eligibility category. The largest categories were Medicaid children (26.4 million), traditional adults (eg, cash assistance, Section 1931, and pregnant women, 11.6 million), expansion adults (those covered under Affordable Care Act expansion categories, 10.9 million), enrollees who are disabled (7.6 million), adults aged 65 years or older (6.4 million), and children enrolled in CHIP (2.3 million). eTable 1 in the Supplement provides more detailed information about characteristics of the Medicaid enrollees.

Our 2 primary measures of Medicaid continuity are the average number of months enrolled in 2016 and the probability of churning during the year, defined as having a break between 2 periods of enrollment. Because the data were a snapshot for calendar year 2016, some enrolled in January were enrolled for some period in 2015, just as some enrolled in December were enrolled afterward in 2017, so the number of months may be left- and/or right-censored.

Aged and disabled enrollees had stable enrollment, averaging over 11 months in the year and with less than 2% probability of churning. Children, whether enrolled in Medicaid or CHIP, had slightly less stable coverage, close to 10 months on average, although children enrolled in CHIP had a higher risk of churning than children with Medicaid (8.0% vs 3.4%). Nondisabled, nonelderly adults had the shortest average enrollment; those covered under expansions averaged 9.8 months, whereas those under traditional Medicaid categories averaged 9.5 months, with 4.2% and 3.8% probabilities of churning, respectively.

Because the 2 adult categories were similar, we combined them and found that the average number of months enrolled was 9.6 months (95% CI, 9.6-9.6). The combined probability of churning in 2016 was 4.0% (95% CI, 4.0%-4.1%). Some 59% of adults were enrolled all 12 months, 10.9% for 1 to 3 months, 10.4% for 4 to 6 months, 10.9% for 7 to 9 months, and 8.3% for 10 to 11 months.

Another issue related to continuity is the enrollment in Medicaid managed care organizations (MCOs). Most Medicaid beneficiaries are enrolled in MCOs, which are responsible for organizing and paying for care for their members and which form provider networks. eTable 2 in the Supplement provides information about the duration of MCO enrollment.

Table 2, Table 3, and Table 4 focus on the enrollment of nondisabled, nonelderly adults in traditional Medicaid and expansion categories, who have the least stable enrollment. Table 2 shows there were surprisingly wide gaps in the average number of months of Medicaid enrollment across the states, ranging from 7.0 months in Texas to 11.2 in Missouri, over 4 months longer.

**Table 3.** Regression Model of Factors Associated With the Number of Months Enrolled in Medicaid in 2016 for Nonelderly, Nondisabled Adults (Weighted, Clustered by State)

Variable	Coefficent (95% CI)
Race and ethnicity	
Black	0.25 (0.13 to 0.36)^a^
Latino	−0.10 (−0.29 to 0.10)
Other, Asian, Native American	0.00 (−0.16 to 0.16)
White	1 [Reference]
Unknown	−0.64 (−0.92 to −0.35)^a^
Female sex	0.38 (0.23 to 0.53)^a^
Age category, y	
18-24	1 [Reference]
25-34	−0.20 (−0.31 to −0.09^a^
35-44	0.03 (−0.11 to 0.17)
45-54	0.09 (−0.12 to 0.31)
55-64	0.11 (−0.25 to 0.47)
Expansion/ex parte	
Both no	1 [Reference]
Expand, not ex parte	0.31 (0.03 to 0.59)^a^
Ex parte, not expand	−3.06 (−3.35 to −2.77)^a^
Both expand and ex parte	−0.18 (−0.44 to 0.09)

^a^ Also controlled for state fixed effects; see eAppendix in the Supplement, r^2^ = .06.

**Table 4.** Logistic Regression Model of Factors Associated With a Break in Medicaid Coverage in 2016 for Nonelderly, Nondisabled Adults (Weighted, Clustered by State)

Variable	Odds ratio (95% CI)
Race and ethnicity	
Black	1.30 (1.23-1.38)^a^
Latino	1.18 (1.09-1.28)^a^
White	1 [Reference]
Other, Asian, Native American	0.98 (0.88-1.10)
Unknown	0.80 (0.68-0.93)
Female sex	1.13 (1.06-1.21)^a^
Age category, y	
18-24	1 [Reference]
25-34	0.79 (0.72-0.86)^a^
35-44	0.68 (0.59-0.78)^a^
45-54	0.61 (0.53-0.69)^a^
55-64	0.49 (0.42-0.58)^a^
Expansion/ex parte	
Both no	1 [Reference]
Expand, not ex parte	0.40 (0.39-0.40)^a^
Ex parte, not expand	0.68 (0.66-0.70)^a^
Both expand and ex parte	0.83 (0.82-0.85)^a^

^a^ Also controlled for state fixed effects; see eAppendix in the Supplement.

### Multivariable Analyses

Ordinary least squares regression models were used to examine demographic and policy or procedural factors that might affect the adults’ enrollment. The regression coefficients effectively measured effects of independent variables on the average number of months enrolled. As seen in Table 3, Black enrollees had slightly longer enrollment than White enrollees, after controlling for other factors. The average duration for women was 0.4 months (95% CI, 0.2-0.5) longer than for men. Those aged 25 to 34 years had slightly shorter enrollment than those aged 18 to 24 years, but older ages did not differ significantly. For brevity, the coefficients for the state fixed effects are not shown in Table 3 or Table 4, but are fully presented in the eAppendix in the Supplement. The state coefficients indicate there are often large interstate differences, not explained by the demographic or policy variables included in the models.

We also examined the interaction between Medicaid expansion and using ex parte reviews*.* Those in Medicaid expansion states without ex parte reviews had an average of 0.31 (95% CI, 0.03-0.59) more months of enrollment than nonexpansion states that did not use ex parte reviews. However, nonexpansion states that used ex parte reviews had shorter average enrollment (−3.06; 95% CI, −3.35 to 2.77 months) than the nonexpansion states not using ex parte reviews. When states both expanded Medicaid and used ex parte reviews, there was no significant difference from the group with neither policy.

Table 4 shows the results of a multivariable logistic regression model that examined factors associated with churning in enrollment, a measure of disruptions between periods of coverage. Black and Latino adults had higher odds of churning than White adults, whereas those with unknown race had lower risk. Women had 13% higher odds of churning (95 CI, 6%-21%) than men. As beneficiaries grew older, the odds of churning consistently declined.

Compared with those living in states without Medicaid expansions or ex parte reviews, those living in states with expansion and no ex parte reviews had 60% lower odds of churning (95% CI, 39%-40%), whereas those in nonexpansion states using ex parte reviews had 68% lower odds (95% CI, 66%-70%). Those in states with both expansion and ex parte reviews had 17% lower odds of churning (95% CI, 15%-18%).

## Discussion

These findings describe factors related to the duration of Medicaid coverage in 2016, before the pandemic-related moratorium on disenrollment. The analyses demonstrate that state policies like expanding Medicaid eligibility or use of ex parte reviews were associated with stability of coverage. Sex, age, and race and ethnicity were also associated with continuity.

These findings are largely consistent with earlier studies^1,8,17^ about the continuity of Medicaid coverage prior to the 2020 moratorium on Medicaid disenrollment. An analysis of 2012 data found that adults had least stable coverage, compared with aged or disabled enrollees, whereas children were in between those 2 categories.^8^ That report also found that continuity varied across states and was lower in some states (eg, Texas) than others (eg, Missouri), suggesting that interstate disparities have persisted for years, perhaps signaling other entrenched operational differences.

This multivariable analyses found that Medicaid expansions were associated with longer average duration of coverage and less churning, although the relationship was complicated by interactions with ex parte reviews. Goldman and Sommers,^21^ using Medical Expenditure Panel Survey data from 2011 to 2016, also found that Medicaid expansion improved continuity of coverage. Fung et al^22^ found that young adults’ insurance continuity improved after Medicaid expansion in Massachusetts. By expanding the income eligibility range, expansions may improve duration because small increases in income are less likely to place a low-income person outside the range of Medicaid eligibility.

A 2021 report by the Medicaid and CHIP Payment and Access Commission (MACPAC) used T-MSIS data from 2017 to 2019 and found that states using higher levels of ex parte reviews had greater continuity.^23^ Our analysis also found that ex parte reviews reduced churning, but were also associated with shorter duration of coverage. These analyses differ in many ways. The MACPAC used more recent data and a 3-year window, but did not examine average duration of enrollment, conduct multivariable analyses, nor examine the role of Medicaid expansions. More states (46) reported use of ex parte in MACPAC’s 2018 data, whereas our 2016 data counted 34 states. Discrepancies in the results might be due to methodological differences or actual changes in state practices.

These analyses found that the relationship of Medicaid expansions and ex parte reviews was complicated. Those in states with Medicaid expansions, in the absence of ex parte, had longer coverage and less churning than those without either policy. Ex parte in nonexpansion states was associated with lower churning, but also shorter average coverage. When both were present, there was no significant association with average duration, but there was lower churning, although the reduction was less than with either policy alone. Taken together, the results indicate that Medicaid expansion both increased average length of enrollment and reduced the risk of churning, and ex parte lowered churning, but also lowered the average length of enrollment.

Medicaid eligibility and ex parte reviews might affect enrollment through different mechanisms. Eligibility expansions broaden the span of income eligibility, bringing more people into coverage so that small income fluctuations do not result in disqualification, which could both lengthen enrollment and reduce churning. The finding that ex parte reviews are associated with both reduced churning and shorter average enrollment duration is surprising. The policy is intended to reduce churning by lowering the need for separate renewal applications, which should reduce beneficiaries’ administrative burdens. In some states, the same automated data capabilities are used for ex parte reviews might also enable them to conduct periodic Medicaid eligibility checks (eg, through wage-matching) during certification periods, which could be used to terminate cases before their certification periods expire and shorten enrollment. Paradoxically, the same automated capabilities that permit ex parte reviews to simplify renewals could also let them conduct periodic eligibility checks that lead to shorter average duration in some states. Not all states conduct periodic eligibility reviews, however, so this problem need not occur in all states.

This study found that race/ethnicity, gender, and age also affect continuity of coverage. Black adults had longer average enrollment, but Black and Latino enrollees had more churning than White enrollees. A recent analysis of Survey of Income and Program Participation data found that Black and Latino enrollees were more likely to be harmed by administrative churning than White enrollees.^13^

This study also found major interstate differences in the average duration of coverage and levels of churning, even after controlling for demographic and policy factors (see eTables 3 and 4 in the Supplement). For example, there was roughly a 4-month difference in average duration of adult coverage in Texas and Missouri in 2016, even though neither expanded Medicaid eligibility and both reported use of ex parte reviews. This suggests that there are other differences in Medicaid enrollment operations that are not well understood, such as more complicated renewal procedures, the relationship between Medicaid and welfare eligibility, or use of periodic checks of eligibility within a certification period. Understanding other operational factors could reveal other approaches to improve continuity of coverage.

In providing guidance to states about the coming Medicaid unwinding, CMS and others have stressed the importance of ex parte reviews in redetermining eligibility.^11,24^ This study found that ex parte reviews were associated with lower churning, but also with shorter average duration of coverage. Future research should more carefully examine how states implement ex parte practices and related state enrollment operations.

After the disenrollment moratorium ends, states can consider policies to stabilize coverage. This analysis suggests that expanding Medicaid is an important option for the 12 states that have not yet expanded their programs under the ACA; it would increase coverage for uninsured people, as well as stabilize coverage for those already enrolled. All states have the option to create 12-month continuous enrollment policies for children, but less than half the states use this option. Research has shown that 12-month continuous enrollment increases the continuity of coverage and is associated with reports of better health.^2,17,25^ Recently, CMS approved a Section 1115 demonstration project waiver in Oregon to continuously enroll children through their sixth birthday.^9^ Section 1115 waivers can also authorize 12-month continuous eligibility for adults; a recent evaluation of New York’s project showed it extended the duration of coverage, increased use of ambulatory care, decreased use of inpatient admissions, and lowered the average monthly cost of Medicaid coverage.^26^ Other states like Illinois, New Jersey, and Oregon have recently proposed to expand Medicaid continuous enrollment for adults.^27^

Data about the effect of the pandemic-related moratorium on disenrollment are still sparse, although administrative data indicate that Medicaid caseloads surged by about one-fifth between early 2020 and early 2022.^28^ One early report^29^ found that caseload increases were caused by higher retention of enrollees, not an influx of new applicants. Another report,^30^ based on preliminary data from an accountable care organization in Massachusetts, showed that enrollment duration had grown longer since 2020 and that the number of people disenrolling had declined. It also found that the change in patterns helped improve enrollment for Black and Hispanic members more than Whites.

Recent projections indicate that between 9 and 16 million people may lose Medicaid coverage after the moratorium ends.^12,13^ Medicaid and CMS advocates have encouraged states take actions to reduce caseload losses, such as updating data about enrollees’ addresses, ensuring that there are sufficient eligibility staff, using ex parte reviews to lower beneficiaries’ administrative burdens and gradually conducting renewals over the 12- to 14-month period. They have also encouraged states to help transition enrollees to other coverage, including plans through health insurance marketplaces.^11,20^ If successful, such actions might reduce the number of people who lose Medicaid and minimize the expected increase in the number of uninsured Americans.

The policies described in this study do not reflect the recent period that began in February 2020, when a temporary moratorium on Medicaid disenrollment began. But it foreshadows what might return when Medicaid unwinding has been completed if states return to their prior policies. Medicaid coverage is likely to become more volatile again, particularly for low-income adults.

### Limitations

A strength of this study is that it is based on a nationally and state-representative data file with a sample of 5.7 million Medicaid enrollees (although data for Arkansas and Tennessee adults were missing). Nonetheless, the data set contains gaps such as missing race and ethnicity data for about 20% of the enrollees. Although enrollees were not duplicated within each state, a person enrolled in one state who moves to another during the year might be counted in both states. Because these analyses were based on administrative data, some information like education, employment, or marital status, were not available. Policy data reported by the Kaiser Family Foundation, such as whether a state used ex parte processing, might be misreported.

An important limitation is that these data are limited to a 12-month window in 2016; many enrolled in January were also enrolled in 2015, just as some enrolled in December continued into 2017, creating a problem of left- and right-censoring. Another study that used a 36-month observation window found patterns like those in this report, although the average duration of enrollment was slightly longer than estimated in this report because it had less censoring.^23^ The 2016 data are not current, although they were just released in June 2022. Finally, these are cross-sectional data, making it difficult to establish that policies like Medicaid expansions or ex parte processing cause the continuity differences.

## Conclusions

In this cross-sectional study of the association of demographic characteristics and state policies with duration of Medicaid coverage and likelihood of churning, policies like expanding Medicaid eligibility and the use of ex parte reviews were associated with less churning and with differences in the average duration of enrollment. But we also found large and puzzling differences in the average duration of coverage across the states that indicate other, deep-seated discrepancies in state policies and operations that may have a substantial effect on Medicaid coverage.

## Notes

Supplement. eAppendix eTable 1. Weighted Distributions of Age, Race/Ethnicity and Gender by Medicaid Eligibility Category, 2016 eTable 2. Weighted Descriptive Data About Number and Percent Enrolled in Medicaid Managed Care Organizations (MCOs), Mean Months of MCO Enrollment Across All Medicaid Enrollees and Among Those Enrolled in MCOs eTable 3. Regression Model of Factors Associated with the Number of Months Enrolled in Medicaid in 2016 for Non-elderly Non-Disabled Adults with State Fixed Effects (weighted, clustered by state) eTable 4. Logistic Regression Model of Factors Associated with a Break in Medicaid Coverage in 2016 for Non-elderly, Non-Disabled Adults with State Fixed Effects (weighted, clustered on state) eReferences
