# Supplementary material for: Duration and Continuity of Medicaid Enrollment Before the COVID-19 Pandemic
Source: JAMA Health Forum. 2022 Dec 16;3(12):e224732. doi: 10.1001/jamahealthforum.2022.4732 (PMC9856506; doi:10.1001/jamahealthforum.2022.4732)
Supplement: Supplement. — eAppendix eTable 1. Weighted Distributions of Age, Race/Ethnicity and Gender by Medicaid Eligibility Category, 2016 eTable 2. Weighted Descriptive Data About Number and Percent Enrolled in Medicaid Managed Care Organizations (MCOs), Mean Months of MCO Enrollment Across All Medicaid Enrollees and Among Those Enrolled in MCOs eTable 3. Regression Model of Factors Associated with the Number of Months Enrolled in Medicaid in 2016 for Non-elderly Non-Disabled Adults with State Fixed Effects (weighted, clustered by state) eTable 4. Logistic Regression Model of Factors Associated with a Break in Medicaid Coverage in 2016 for Non-elderly, Non-Disabled Adults with State Fixed Effects (weighted, clustered on state) eReferences [file jamahealthforum-e224732-s001.pdf]

Supplemental Online Content

Ku L, Platt I. Duration and continuity of Medicaid enrollment before the COVID-19 pandemic. *JAMA Health Forum*. 2022;3(12):e224732. doi:10.1001/jamahealthforum.2022.4732

eAppendix
eTable 1. Weighted Distributions of Age, Race/Ethnicity and Gender by Medicaid Eligibility Category, 2016
eTable 2. Weighted Descriptive Data About Number and Percent Enrolled in Medicaid Managed Care Organizations (MCOs), Mean Months of MCO Enrollment Across All Medicaid Enrollees and Among Those Enrolled in MCOs
eTable 3. Regression Model of Factors Associated with the Number of Months Enrolled in Medicaid in 2016 for Non-elderly Non-Disabled Adults with State Fixed Effects (weighted, clustered by state)
eTable 4. Logistic Regression Model of Factors Associated with a Break in Medicaid Coverage in 2016 for Non-elderly, Non-Disabled Adults with State Fixed Effects (weighted, clustered on state)
eReferences

This supplemental material has been provided by the authors to give readers additional information about their work.

© 2022 Ku L, et al. *JAMA Health Forum.*

eAppendix

The Duration and Continuity of Medicaid Enrollment Before the COVID-19 Pandemic

This supplementary appendix provides more description and additional tables that complement the main text.

Data Notes

The primary data used were the Medicaid person files from the Synthetic Healthcare Data for Research (SyH-DR) for 2016, released by the Agency for Healthcare Research and Quality (AHRQ) in June 2022.[1 2] The data are weighted to represent 69.8 million enrollees nationwide. They are based on samples of data submitted by Medicaid state agencies as part of the Transformed Medicaid Statistical Information System (T-MSIS), which included data from all 50 states and the District of Columbia, except Arkansas. Tennessee data only included children. AHRQ staff view the SyH-DR data as a first step in the development and release of national-level all-payer claims data bases; in addition to providing nationally representative data about Medicaid, it also provides de-identified data for Medicare and commercial health insurance. To protect the privacy of health data, the data are de-identified and sometimes are aggregated and "synthesized" (selectively scrambled) as described in the documentation. We believe the specific data we used were not synthesized and reflect a direct sample of T-MSIS data. The SyH-DR data do not contain as much detail as the original claims files.

We found a small number of data problems and cleaned them where possible. For example, we found 2,876 cases out of the 5.7 million sampled cases where the person has zero months of Medicaid enrollment and we eliminated them. In a few cases, we found age data that appeared inconsistent with the ages that should have been possible for their eligibility category, e.g., children under 18 in the expansion adult category; we eliminated these as well. The biggest gap was that 19.5% of enrollees had "unknown" race. It is well known that reporting of race/ethnicity in Medicaid is often incomplete; we included those cases and made Unknown as a separate category of race/ethnicity. It is not clear from the documentation whether race/ethnicity was self-reported by enrollees or determined by staff; it is plausible that both methods are used.

Data about state policies for 2016 came from the Kaiser Family Foundation (KFF), which surveyed state Medicaid agencies about their enrollment policies.[3] Of particular importance, we used data about *ex parte* reviews in renewing Medicaid eligibility, which means that agencies look at other data available to them to conduct renewals. For example, if a person's Medicaid certification period expires in March, but SNAP eligibility was determined in January, the SNAP data can be used to renew their coverage and there is no need to ask for a renewal application. Federal regulations require the use of *ex parte* reviews for those whose incomes is based on Modified Adjusted Gross Income (MAGI) criteria, but the KFF data indicated that only 34 states reported using it in 2016. KFF also asked what percent of renewals were "completed via *ex parte"* (< 25%, 25%-50%, 50%-75%, and 75%+) but 8 of the 34 states did not have an estimated percentage. We were unsure about how to interpret the percentages for four reasons: (1) Not every renewal is a MAGI case (e.g., aged, disabled and foster enrollees are typically not MAGI) and it was not clear if these cases were in the denominator or not. (2) Medicaid enrollment is typically

© 2022 Ku L, et al. *JAMA Health Forum.*

much larger than SNAP or TANF enrollment and we would not expect all (or even a majority) of Medicaid enrollees to have alternative data available. Some states may use wage matching data from unemployment systems, but not all do and wage-matching data are often incomplete and not current. (3) Does having a renewal completed vi*a ex parte* mean that the other information led to a renewal or that a check was completed, even if they found no other data and then asked the person to submit a renewal form? (4) In principle, given the federal regulation, all states are supposed to use *ex parte* reviews. Did the fact that 17 states did not report *ex parte* mean they are not doing it or that it was not reported to KFF? Given this uncertainty, we just used *ex parte* reviews as a binary variable of used *ex parte* or not. In contrast, a recent MACPAC report used the percentage of *ex parte* reviews using 2018 KFF data in which 46 states reported using *ex parte* reviews and found that greater use of ex parte was associated with longer continuity, although it did not test for statistical significance nor controlled for other demographic and policy factors.[4]

Enrollee Demographic Characteristics

There were a limited set of data about enrollees. **Table A-1** provides a tabulation of the percentage of enrollees in each of six Medicaid eligibility characteristics by age category, race/ethnicity and gender, in states that expanded Medicaid as of January 1, 2016 and those that did not. Expansion states had a larger percentage of enrollees who were adults 18 to 64 years old than non-expansion states and therefore a smaller percentage who were children 0 to 17. Expansion states had a higher percentage of enrollees who were Latino, White or Other, while non-expansion states had higher percentage of enrollees who were Black or Unknown race/ethnicity. Expansion and non-expansion states had similar gender patterns, although traditional adult enrollees were more likely to be female in both expansion and non-expansion states.

Medicaid Managed Care Organization Enrollment

Over two-thirds of Medicaid enrollees receive care from Medicaid managed care organizations (MCOs), which are responsible for the organization and delivery of health services for their members (although some Medicaid services or medications may be "carved out" and still under fee-for-service systems). The MCOs determine the networks of providers who are available for care for their members, although sometimes members may see out-of-network providers. Some Medicaid enrollees are not in MCOs because the state has not established Medicaid MCOs for some categories (e.g., the aged) or because some are participating in another form of managed care, Primary Care Case Management, which does not require MCO membership.

Table A-2 provides information about MCO membership and the average duration of MCO enrollment by eligibility category. We define MCO membership as having at least one month enrolled in an MCO. As can be seen, Medicaid children and expansion adults are the most likely to be enrolled in MCOs (93% and 88%, respectively), followed by CHIP children, traditional adults and people with disabilities (75%, 74% and 73%, respectively). Aged beneficiaries, almost all of whom are dually enrolled in Medicare, are least likely to be in Medicaid MCOs (50%).

The average months of MCO membership is displayed in two ways. First is the average number of months enrolled in MCOs, using all Medicaid enrollees as the denominator. By this measure, the average span in an MCO is consistently shorter than the average months of enrollment

© 2022 Ku L, et al. *JAMA Health Forum.*

shown in Table 1, between 4.9 and 8.7 months enrolled. But when the average duration is computed only for those with at least one month of MCO membership, the average increases to between 8.4 and 10.0 months, still slightly lower than the overall average number of months enrolled, as seen in Table 1.

One reason the duration of enrollment in MCOs is shorter is that new Medicaid enrollees are not automatically enrolled in MCOs, but are usually given one or two months to select an MCO (if one is available for them). Thus, they first spend on two months in fee-for-service care prior to enrollment in an MCO. If they do not make a selection, they are auto-enrolled in an MCO selected by the state agency, although they typically have a grace period to switch plans if they wish. When they renew coverage, the state may automatically assign them to the same MCO they had, offer them an option to change plans or auto-assign them if no choice is made. The somewhat briefer time spent in an MCO affects the delivery and financing of care in Medicaid, since they spent less time covered by the MCO and participating in the MCO's provider network. MCOs incur some administrative costs on-boarding new enrollees, so this also affect MCO finances.[5]

Multivariate Analyses with State Fixed Effects

As noted in the main text, our multivariate models of factors associated with the number of months enrolled in Medicaid and with having breaks in coverage incorporated state fixed effects, implemented by adding a binary dummy variable for every state except Texas. Adding the fixed effects helps control for other unmeasured state characteristics that might also affect Medicaid continuity. Texas was selected as the omitted reference state since initial analysis showed it had the lowest average duration among the states.

The coefficients for the state fixed effects are shown in **Tables A-3** and **A-4**, which correspond with Tables 3 and 4 in the main text but add the state-level coefficients. Fixed effects coefficients could not be estimated for a few states. As seen in Tables A-3 and A-4, coefficients for Vermont, West Virginia and Wisconsin could not be estimated due to collinearity between the policy variables and the other state IDs and policy variables. In Table A-4, the model about breaks in coverage, three more states (Kentucky, New York and Wyoming) were not included because they each reported no breaks in coverage. Nonetheless, we could estimate coefficients for state fixed effects for most variables.

A noteworthy finding is that, even after controlling for demographic and state policy variables, there were large and usually highly significant coefficients or odds ratios for the state fixed effects. For example, in Table A-3, the coefficient for Missouri (4.1, 95% CI = 4.0 to 4.2) means that even after controlling for the demographic and policy variables, Missouri's average enrollment duration is about 4 months longer than that of Texas, the omitted reference state. This is very close to the unadjusted difference of 4 months between the Missouri and Texas in Table 2. But while Texas had the lowest unadjusted average number of months in Table 2, the ordinary least squares regression model in Table A-3 indicates that some states like Georgia or North Dakota have even lower duration than Texas, after controlling for other factors.

© 2022 Ku L, et al. *JAMA Health Forum.*

The odds ratios for the state fixed effects in Table A-4 indicate that most states have a lower odds of breaks in coverage than Texas, the omitted reference state, after controlling for the demographic and state policy variables.

The presence of large, unexplained differences in the average duration of Medicaid enrollment and the odds of coverage breaks across the states suggest that there could be other unmeasured demographic, policy or operational factors that contribute to large interstate differences in the continuity of enrollment in Medicaid. Our policy variables included state Medicaid expansions and *ex parte* reviews, although we also tested the association of telephone renewals and pre-populated renewal forms with continuity of coverage, but did not find significant associations. We could not control for immigration status, employment, health status, marital status or the presence of children in the family because those variables were not available the SyH-DR data. There may also be other policy factors, such as the length or complexity of the renewal process, the extent to which states had current correct addresses for their enrollees, or the availability of staff to help applicants complete renewals that might also affect the continuity of enrollment.

© 2022 Ku L, et al. *JAMA Health Forum.*

eTable 1. Weighted Distributions of Age, Race/Ethnicity and Gender by Medicaid Eligibility Category, 2016

	Age Categories (in Years)				
**Expansion States**	**0-5**	**6-17**	**18-34**	**35-64**	**65+**
Medicaid children	38.1%	61.9%	0.0%	0.0%	0.0%
CHIP children	25.9%	74.1%	0.0%	0.0%	0.0%
Traditional adults	0.0%	0.0%	61.3%	38.7%	0.0%
Disabled	3.5%	13.7%	18.4%	64.0%	0.5%
Aged	0.0%	0.0%	0.0%	0.0%	100.0%
Expansion adults	0.0%	0.0%	45.9%	51.9%	1.1%
**Overall**	**15.3%**	**26.5%**	**23.4%**	**25.0%**	**9.8%**
**Non-Expansion States**					
Medicaid children	41.1%	58.9%	0.0%	0.0%	0.0%
CHIP children	21.3%	78.7%	0.0%	0.0%	0.0%
Traditional adults	0.0%	0.0%	71.9%	28.1%	0.0%
Disabled	7.5%	14.5%	19.0%	58.8%	0.2%
Aged	0.0%	0.0%	0.0%	0.0%	100.0%
Expansion adults	0.0%	0.0%	47.6%	52.2%	0.1%
**Overall**	**21.2%**	**33.4%**	**18.1%**	**16.7%**	**10.6%**

	Racial/Ethnic Category				
	**Black**	**Latino**	**White**	**Other**	**Unknown**
**Expansion States**					
Medicaid children	16.1%	28.3%	33.2%	7.2%	15.2%
CHIP children	7.8%	12.0%	41.4%	6.3%	32.6%
Traditional adults	15.1%	28.2%	34.0%	8.4%	14.4%
Disabled	21.7%	11.4%	48.8%	4.0%	14.2%
Aged	12.2%	16.5%	43.6%	13.4%	14.3%
Expansion adults	15.5%	17.3%	42.4%	8.5%	16.3%
**Overall**	**15.7%**	**22.6%**	**38.2%**	**8.0%**	**15.6%**
**Non-Expansion States**					
Medicaid children	24.7%	20.4%	31.2%	3.4%	20.3%
CHIP children	16.5%	26.4%	36.0%	5.9%	15.2%
Traditional adults	28.0%	11.4%	40.7%	3.8%	16.1%
Disabled	22.4%	7.2%	32.5%	1.6%	36.3%
Aged	20.7%	10.5%	40.9%	3.5%	24.4%
Expansion adults	31.9%	4.4%	45.3%	2.4%	16.0%
**Overall**	**24.3%**	**15.3%**	**34.9%**	**3.3%**	**22.3%**

	Gender	
	**Male**	**Female**
**Expansion States**		
Medicaid children	50.5%	49.6%
CHIP children	50.9%	49.1%
Traditional adults	30.0%	70.0%
Disabled	51.8%	48.2%
Aged	38.2%	61.8%
Expansion adults	50.0%	50.1%
**Overall**	45.8%	54.2%
**Non-Expansion States**		
Medicaid children	50.2%	49.8%
CHIP children	50.7%	49.3%
Traditional adults	27.3%	72.7%
Disabled	56.0%	44.0%
Aged	37.9%	62.1%
Expansion adults	46.4%	53.6%
**Overall**	45.5%	54.5%

Source: SyH-DR Medicaid person data, 2016

© 2022 Ku L, et al. *JAMA Health Forum.*

eTable 2. Weighted Descriptive Data About Number and Percent Enrolled in Medicaid Managed Care Organizations (MCOs), Mean Months of MCO Enrollment Across All Medicaid Enrollees and Among Those Enrolled in MCOs*

Category	# Enrolled in MCOs*	% Enrolled in MCOs*	Months in MCOs - All Enrollees		Months in MCOs Among Those Enrolled in MCOs	
			Mean	95% Conf. Int.	Mean	95% Conf. Int.
Medicaid Children	24.5 million	92.7%	8.69	8.69 - 8.70	9.38	9.38 - 9.39
CHIP Children	1.7 million	75.1%	6.86	6.84 - 6.89	9.14	9.13 - 9.16
Traditional Adults	8.6 million	74.2%	6.22	6.21 - 6.23	8.39	8.38 - 8.40
Expansion Adults	9.7 million	88.4%	7.50	7.49 - 7.50	8.48	8.47 - 8.49
Disabled	5.6 million	72.7%	7.25	7.24 - 7.26	9.98	9.97 - 9.99
Aged	3.2 million	49.8%	4.86	4.84 - 4.87	9.75	9.73 - 9.76

* Those enrolled in MCOs are defined as those enrolled in an MCO for at least one month in the year

Source: SyH-DR Medicaid person data, 2016

n = 5,403,960

© 2022 Ku L, et al. *JAMA Health Forum.*

eTable 3. Regression Model of Factors Associated with the Number of Months Enrolled in Medicaid in 2016 for Non-elderly Non-Disabled Adults with State Fixed Effects (weighted, clustered by state)

Variable	Coefficent		95% Confidence Int.
**Race/Ethnicity (ref = White)**			
Black	0.25	***	0.13 - 0.36
Latino	-0.10		-0.29 - 0.10
Other, Asian, Nat Amer	0.00		-0.16 - 0.16
Unknown	-0.64	***	-0.92 - -0.35
**Female**	0.38	***	0.23 - 0.53
**Age Category (ref = 18-24)**			
25-34	-0.20	***	-0.31 - -0.09
35-44	0.03		-0.11 - 0.17
45-54	0.09		-0.12 - 0.31
55-64	0.11		-0.25 - 0.47
**Expansion/Ex Parte (Ref = both no)**			-
Expand, Not Ex Parte	0.31	*	0.03 - 0.59
Ex Parte, Not Expand	-3.06	***	-3.35 -2.77
Both Expand & Ex Parte	-0.18		-0.44 0.09
**State Fixed Effect (ref = Texas)**			
Alaska	-0.79	***	-0.87 - -0.70
Alabama	-1.40	***	-1.64 - -1.16
Arizona	0.96	***	0.93 - 0.99
California	-0.21	***	-0.33 - -0.09
Colorado	0.07		-0.01 - 0.16
Connecticut	0.22	***	0.17 - 0.27
District Columbia	0.23	***	0.15 - 0.31
Delaware	-1.80	***	-1.88 - -1.72
Florida	3.20	***	3.07 - 3.32
Georgia	-2.42	***	-2.68 - -2.16
Hawaii	0.97	***	0.88 - 1.06
Idaho	-0.85	***	-0.99 - -0.71
Iowa	1.53	***	1.43 - 1.63
Illinois	-0.58	***	-0.63 - -0.53
Indiana	-1.45	***	-1.50 - -1.41
Kansas	2.36	***	2.30 - 2.42
Kentucky	1.07	***	1.05 - 1.09
Louisiana	1.94	***	1.85 - 2.03
Massachusetts	-0.31	***	-0.40 - -0.23
Maryland	0.00		-0.05 - 0.05
Maine	-0.56	***	-0.85 - -0.27
Michigan	-0.86	***	-0.89 - -0.83
Minnesota	-0.93	***	-0.95 - -0.90
Missouri	4.12	***	4.04 - 4.20
Mississippi	-0.81	***	-0.90 - -0.71
Montana	-1.43	***	-1.47 - -1.39
North Carolina	3.12	***	3.04 - 3.19
North Dakota	-2.14	***	-2.21 - -2.08
Nebraska	1.79	***	1.50 - 2.09
New Hampshire	-1.08	***	-1.11 - -1.04
New Jersey	-0.39	***	-0.45 - -0.33
New Mexico	0.44	***	0.30 - 0.58
Nevada	-1.63	***	-1.71 - -1.56
New York	0.86	***	0.81 - 0.91
Ohio	0.68	***	0.64 - 0.71
Oklahoma	0.92	***	0.86 - 0.98
Oregon	0.52	***	0.45 - 0.58
Pennsylvania	-0.81	***	-0.86 - -0.75
Rhode Island	0.79	***	0.57 - 1.02
Sourth Carolina	3.49	***	3.39 - 3.59
South Dakota	0.80	***	0.73 - 0.87
Utah	0.75	***	0.66 - 0.84
Virginia	1.92	***	1.83 - 2.00
Vermont	omitted due to collineariy		-
Washington	-0.08	*	-0.16 - -0.01
Wisconsin	-1.01	***	-1.30 - -0.72
West Virginia	omitted due to collineariy		
Wyoming	omitted due to collineariy		
**Constant**	9.87	***	9.57 - 10.18

* p < .05; ** p < .01; *** p < .001

Source: SyH-DR Medicaid person data, 2016

n = 2,173,527 r^2 = .0624

© 2022 Ku L, et al. *JAMA Health Forum.*

eTable 4. Logistic Regression Model of Factors Associated with a Break in Medicaid Coverage in 2016 for Non-elderly, Non-Disabled Adults with State Fixed Effects (weighted, clustered on state)

Variable	Odds Ratio	95% Confidence Int.
**Race/Ethnicity (ref = White)**		
Black	1.30 ***	1.23 - 1.38
Latino	1.18 ***	1.09 - 1.28
Other, Asian, Nat Amer	0.98	0.88 - 1.10
Unknown	0.80	0.68 - 0.93
**Female**	1.13 ***	1.06 - 1.21
**Age Category (ref = 18-24)**		-
25-34	0.79 ***	0.72 - 0.86
35-44	0.68 ***	0.59 - 0.78
45-54	0.61 ***	0.53 - 0.69
55-64	0.49 ***	0.42 - 0.58
**Expansion/Ex Parte (Ref = both no)**		-
Expand, Not Ex Parte	0.40 ***	0.39 - 0.40
Ex Parte, Not Expand	0.68 ***	0.66 - 0.70
Both Expand & Ex Parte	0.83 ***	0.82 - 0.85
		-
**State Fixed Effect (ref = Texas)**		-
Alaska	1.20 ***	1.14 - 1.26
Alabama	0.26 ***	0.26 - 0.27
Arizona	0.02 ***	0.02 - 0.02
California	0.45 ***	0.43 - 0.47
Colorado	0.52 ***	0.51 - 0.54
Connecticut	0.73 ***	0.71 - 0.74
District Columbia	0.48 ***	0.45 - 0.50
Delaware	0.80 ***	0.77 - 0.83
Florida	0.00 ***	0.00 - 0.00
Georgia	0.56 ***	0.55 - 0.58
Hawaii	0.02 ***	0.02 - 0.02
Idaho	1.22 ***	1.16 - 1.28
Iowa	0.35 ***	0.34 - 0.37
Illinois	0.78 ***	0.76 - 0.80
Indiana	1.00	0.98 - 1.02
Kansas	0.19 ***	0.18 - 0.19
Kentucky	(omitted because no breaks)	
Louisiana	0.38 ***	0.37 - 0.40
Massachusetts	1.04	0.99 - 1.09
Maryland	0.35 ***	0.34 - 0.36
Maine	0.65 ***	0.64 - 0.66
Michigan	1.81 ***	1.77 - 1.84
Minnesota	0.70 ***	0.69 - 0.71
Missouri	0.21 ***	0.21 - 0.22
Mississippi	0.30 ***	0.28 - 0.33
Montana	1.08 ***	1.06 - 1.11
North Carolina	0.30 ***	0.29 - 0.32
North Dakota	0.54 ***	0.52 - 0.56
Nebraska	0.91	0.78 - 1.05
New Hampshire	0.67 ***	0.66 - 0.68
New Jersey	0.48 ***	0.47 - 0.49
New Mexico	0.43 ***	0.42 - 0.45
Nevada	1.57 ***	1.53 - 1.61
New York	(omitted because no breaks)	
Ohio	0.13 ***	0.13 - 0.14
Oklahoma	1.02	0.98 - 1.06
Oregon	0.20 ***	0.19 - 0.20
Pennsylvania	0.78 ***	0.76 - 0.80
Rhode Island	0.66 ***	0.58 - 0.75
Sourth Carolina	0.19 ***	0.18 - 0.20
South Dakota	0.91 **	0.86 - 0.97
Utah	0.95 **	0.92 - 0.99
Virginia	0.66 ***	0.64 - 0.69
Vermont	(omitted due to collineariy)	
Washington	0.36 ***	0.36 - 0.37
Wisconsin	(omitted due to collineariy)	
West Virginia	(omitted due to collineariy)	
Wyoming	(omitted because no breaks)	
**Constant**	0.14 ***	0.12 - 0.15

* p < .05; ** p < .01; *** p < .001

Source: SyH-DR Medicaid person data, 2016

n = 1,908,879 r^2 = .0548

© 2022 Ku L, et al. *JAMA Health Forum.*

eReferences

[1] Agency for Healthcare Research and Quality. Synthetic Healthcare Database for Research (SyH-DR). June 2022. https://www.ahrq.gov/data/syh-dr.html. Accessed June 17, 2022.

[2] Agency for Healthcare Research and Quality. Synthetic Healthcare Database for Research (SyH-DR) A Synthetic Nationally Representative All-Payer Claims Database: Sampling, Weighting, and Synthetization Methodologies. AHRQ Publication No. 22-0039-2-EF. June 2022. https://www.ahrq.gov/sites/default/files/wysiwyg/data/SyH-DR-Sampling-Weighting-Synthetization-Methodologies-rev.pdf. Accessed June 17, 2022.

[3] Brooks T, Miskell S, Artiga S, et al. Medicaid and CHIP Eligibility, Enrollment, Renewal, and Cost-Sharing Policies as of January 2016: Findings from a 50-State Survey. Jan. 2016. https://www.kff.org/medicaid/report/medicaid-and-chip-eligibility-enrollment-renewal-and-cost-sharing-policies-as-of-january-2016-findings-from-a-50-state-survey/. Accessed June 17, 2022.

[4] Medicaid and CHIP Payment and Access Commission. An Updated Look at Rates of Churn and Continuous Coverage in Medicaid and CHIP. Oct. 2021. https://www.macpac.gov/publication/an-updated-look-at-rates-of-churn-and-continuous-coverage-in-medicaid-and-chip-abstract/. Accessed June 29, 2022.

[5] Fairbrother G, Dutton MJ, Bachrach D, Newell, K.A., Boozang, P., Cooper, R. “Costs of enrolling children in Medicaid and SCHIP.” *Health Affairs.* 2004; 23(1):237-43.

© 2022 Ku L, et al. *JAMA Health Forum.*
